# Preparation and Research of Monolayer WS_2_ FETs Encapsulated by h-BN Material

**DOI:** 10.3390/mi12091006

**Published:** 2021-08-24

**Authors:** Tao Han, Hongxia Liu, Shupeng Chen, Shulong Wang, Kun Yang

**Affiliations:** Key Laboratory for Wide-Band Gap Semiconductor Materials and Devices of Education, The School of Microelectronics, Xidian University, Xi’an 710071, China; 15639119745@163.com (T.H.); slwang@xidian.edu.cn (S.W.); kuny2019@163.com (K.Y.)

**Keywords:** WS_2_, h-BN, vdWs heterostructure, characterization, FET

## Abstract

Functional devices that use vertical van der Waals (vdWs) heterostructure material can effectively combine the properties of single component materials, and the strong interlayer coupling effect can change their electronic and optical properties. According to our research, WS_2_/h-BN vertical vdWs heterostructure material can be synthesized by chemical vapor deposition (CVD) and wet transfer methods. Monolayer WS_2_ material and WS_2_/h-BN vertical vdWs heterostructure material can be tested and characterized using XPS, SEM, EDS, AFM and Raman spectroscopy, which can prove the existence of corresponding materials. When the thickness of the material decreases, the Coulomb scattering amongst two-dimensional (2D) layered materials increases. This is because both the shielding effect and the distance between the channel and the interface layer decrease. FET devices are then fabricated on WS_2_/h-BN vdWs heterostructure material by the electron beam lithography and evaporation processes. The effects of vdWs epitaxy on electrical transmission when WS_2_/h-BN vdWs heterostructure material is formed are explored. Finally, the related electrical performance of FET devices is tested and analyzed. Our experimental research provides guidance for the use of electronic devices with vdWs heterostructure material.

## 1. Introduction

Silicon-based transistors encounter short-channel effects and drain-induced barrier reduction problems as a device decreases in size [1,2]. To overcome these problems, atomic thickness channel material is used to eliminate the potential adverse effects of device miniaturization. Monolayer WS_2_ material has high mobility and a direct band gap of 2 eV, while also exhibiting valley polarization [3]. Therefore, monolayer WS_2_ and heterostructure materials are important for basic research and device applications, such as for photodetectors [4] and field effect transistors (FETs) [5]. The band gap of thin-layer hexagonal boron nitride (h-BN) material is 5.97 eV. The h-BN material also has a hexagonal lattice structure on the 2D plane, and the ionic bond in the layer is strong. Therefore, the chemical properties of h-BN material are stable [6,7,8]. There are no dangling bonds on the surface of h-BN material, which helps to decrease surface effects and electron scattering and improves mobility. The h-BN material is used as the encapsulation layer, which has no effect on the performance of underlying material [9]. As an excellent semiconductor material, WS_2_/h-BN vertical vdWs heterostructure material can be used for 2D ultra-thin electronics [10] and optoelectronic devices [11]. 

The preparation of WS_2_/h-BN vertical vdWs heterostructure material mainly includes chemical vapor deposition (CVD) preparation, i.e., mechanical peeling and transfer stacking methods [12,13]. However, the mechanical peeling and stacking method is not suitable for large-scale fabrication due to its lower yield [14]. During the transfer process, the transfer stacking method introduces impurity contamination between the h-BN and the WS_2_ material interfaces, which has a marked impact on the properties of WS_2_/h-BN heterostructure material [15]. The preparation of large-area, controllable, continuous and uniform WS_2_/h-BN heterostructure material is still the eminent challenge. In addition, h-BN material can be used as a packaging material, which can decrease surface chemical adsorption and the influence of the external environment on the channel material [16,17]. To improve the performance of nanodevices, different vdWs vertical heterostructure materials can be formed by h-BN, graphene and TMDs materials. Therefore, the controllable preparation of WS_2_/h-BN heterostructure vertical material has great significance. 

Our research mainly consists of the following aspects: First, WS_2_, h-BN and WS_2_/h-BN vdWs vertical heterostructure materials are grown and prepared by CVD and wet transfer methods. Next, monolayer WS_2_ material is characterized by x-ray photoelectron spectroscopy (XPS), an atomic force microscope (AFM), scanning electron microscopy (SEM) and energy dispersive x-ray (EDX) spectroscopy. The Raman spectrometer is also used to test and characterize the spectral characteristics of WS_2_/h-BN heterostructure material in order to explore the influence of vdWs epitaxy. Subsequently, the field effect transistor (FET) based on WS_2_/h-BN vdWs vertical heterostructure material is prepared. Finally, the electronic transmission performance of FET devices is measured and analyzed, and the channel characteristics are enhanced by h-BN material, which is achieved by reducing external defects. 

## 2. Materials and Methods

### 2.1. Preparation of WS_2_ Material

Monolayer WS_2_ material was prepared using the CVD method. During the reaction process of the WS_2_ material, the film quality was precisely controlled by adjusting the heating temperature, gas flow rate and reaction time [18]. This method has the advantages of large film size, high crystallinity and precise controllability. High-purity WO_3_ and sulfur powders were selected as the tungsten source and sulfur source, respectively. WO_3_ and sulfur powders (SixCarbon Technology Shenzhen, Shenzhen, China) were placed in the different reaction temperature zones of the tube furnace, and SiO_2_/Si substrate was placed 7 cm below the WO_3_ powder. To achieve the sublimation of the WO_3_ powder and sulfur powder, the temperature of the powders was set to 1000 °C and 200 °C, respectively. The WO_3_ and sulfur vapors diffused into the SiO_2_/Si substrate with the help of 50 sccm of Ar gas and reacted for 10 min, and thus WS_2_ material was obtained.

### 2.2. Preparation of h-BN Material

Monolayer h-BN film was grown on a copper foil substrate by a tube furnace, and the Borane Ammonia Complex was used as the growth source [19]. First, the copper foil substrate and 100 mg of borane ammonia complex were placed on the high temperature zone and the upper end of the tube furnace, respectively. The high temperature zone of the tube furnace was then heated to 1050 °C, and 10 sccm of hydrogen (H_2_) gas and 100 sccm of argon (Ar) gas were continuously introduced during the heating process. Next, the copper foil substrate was annealed at 1050 °C for 1 h. Immediately after, the borane ammonia complex was heated at 100 °C, which resulted in the production of nitrogen and boron. The growth time was maintained for 20 min, during which time B and N atoms combined to form h-BN material on the copper foil substrate. Finally, the copper foil with the h-BN film was removed when the high temperature zone was allowed to cool naturally.

### 2.3. Preparation of WS_2_/h-BN vdWs Vertical Heterostructure Material

To begin with, the PMMA solution was uniformly spin coated on the copper foil substrate covered with h-BN material at 500 rpm and 2500 rpm for 10 s and 20 s, respectively. Then, this copper foil substrate, spin-coated with the PMMA solution, was placed in a 120 °C oven for 3 min to cure the PMMA film, from which the PMMA/h-BN/copper foil substrate can be obtained. The PMMA/h-BN/copper foil substrate was then immersed in 1M FeCl_3_ solution for 1 h to corrode the copper foil and make the PMMA/h-BN film float on the surface of the solution. Next, the PMMA/h-BN film was washed with deionized water several times to remove the residual FeCl_3_ solution, and then the PMMA/h-BN film was placed on the WS_2_/SiO_2_/Si substrate. The PMMA/h-BN/WS_2_/SiO_2_/Si substrate was then placed at 90 °C for 1h to remove any water, which allows the PMMA/h-BN film to bond more tightly to the target substrate [20]. Finally, the PMMA layer was dissolved in a 40 °C hot acetone solution. The h-BN/WS_2_/SiO_2_/Si target substrate was rinsed with isopropanol solution and then blown dry with high-purity nitrogen (N_2_) gas.

### 2.4. Characterization Methods

The morphology, composition, layer thickness and structural properties of WS_2_/h-BN vdWs vertical heterostructure material can be characterized using XPS, SEM, AFM and Raman spectrometers. The Raman and PL spectra of WS_2_/h-BN heterostructure material were tested by Horiba LabRAM HR equipment (Jobin Yvon, France). A 532 nm laser equipped with 1800 g/mm grating can be selected in the Raman spectrometer, which can analyze the layers’ number, crystallinity and band gap [21]. The incident laser power is at 20 μW, the resolution is greater than 1 cm^2^/pixel and the laser spot diameter is about 1 μm. Raman and PL spectrum data were acquired and analyzed by LabSpec 5 and Origin 16 software, respectively. AFM is used to characterize the surface morphology and thickness, and can accurately identify the number of nanomaterials in the layers by combining Raman and photoluminescence spectroscopy with optical contrast [22]. The morphology and surface structure of WS_2_/h-BN vdWs vertical heterostructure material was characterized by SEM [23]. XPS can also be used to characterize the surface elements and chemical structure of material [24].

## 3. Results and Discussion

### 3.1. Test Characterization of WS_2_ Material

**Figure 1:**
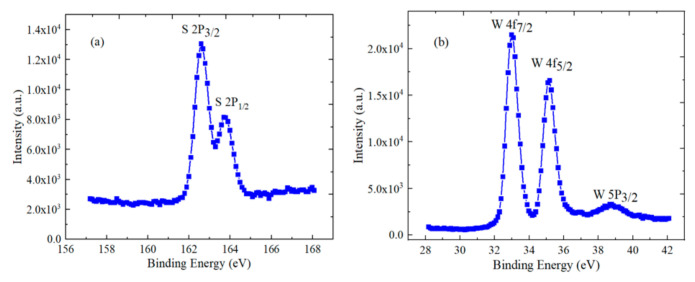
XPS spectra of WS_2_ material (**a**) S element, (**b**) W element.

The element composition and valence state of WS_2_ material were analyzed by XPS, using the ESCALAB250 (Thermo Scientific Company, Waltham, MA, USA) instrument. During the process of characterization, the monochromatic Al Kα rays (1486.6 eV) were used as a radiation source, and all binding energies were measured and calibrated with the C 1s peak (284.6 eV) of carbon [25]. Observation of Figure 1a shows that 2P_3/2_ and 2P_1/2_ of S element are located at 162.57 eV and 163.67 eV, respectively. In Figure 1b, 4f_7/2_, 4f_5/2_ and 5P_3/2_ of W element are at 32.97 eV, 35.17 eV and 38.67 eV, respectively. The bond energies of W and S elements are consistent with the previous reports, which can prove the existence of WS_2_ material.

**Figure 2:**
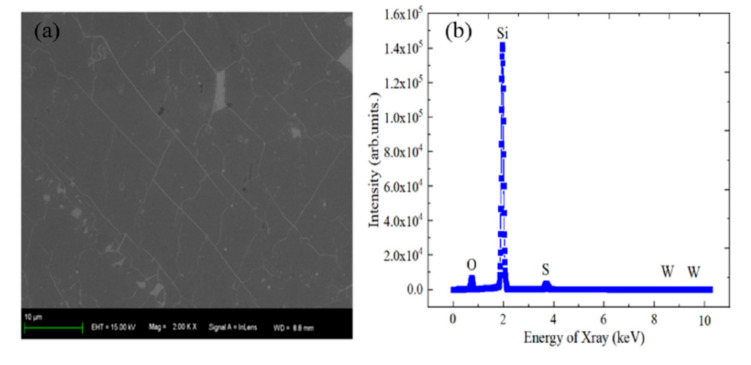
(**a**) SEM image and (**b**) EDX spectrum of WS_2_ material.

The backscattered and secondary electron signals can be obtained when high-energy electron beams hit the sample surface. The surface morphology of material can be mastered by the amplification and analysis of the above signals [26]. The surface and section morphology of WS_2_ material can be observed by SEM. Figure 2a shows that WS_2_ material has good film-forming properties. W and S elements do exist on SiO_2_/Si substrate, as shown in Figure 2b, where the composition of WS_2_ material was characterized by an energy spectrum test.

**Figure 3:**
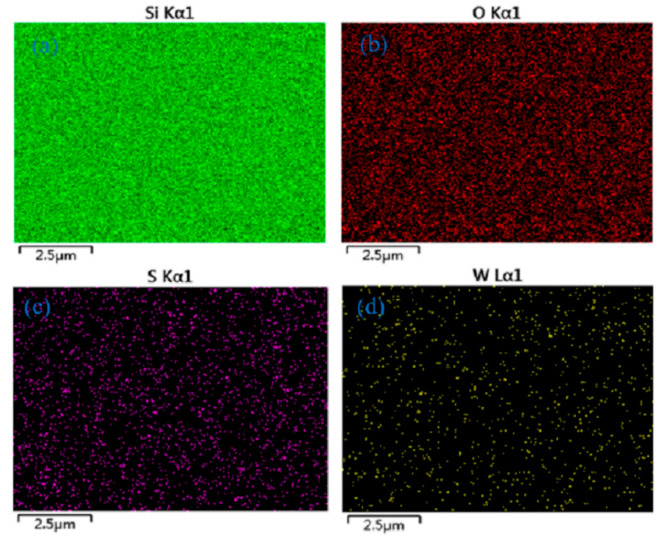
EDS element imaging of WS_2_ material on SiO_2_/Si substrate. (**a**) Si, (**b**) O, (**c**) S, (**d**) W.

Observing Figure 3 reveals that the uniform distribution of W and S elements on the SiO_2_/Si substrate can be obtained by the planar EDS imaging, which further proves the existence of WS_2_ material.

**Figure 4:**
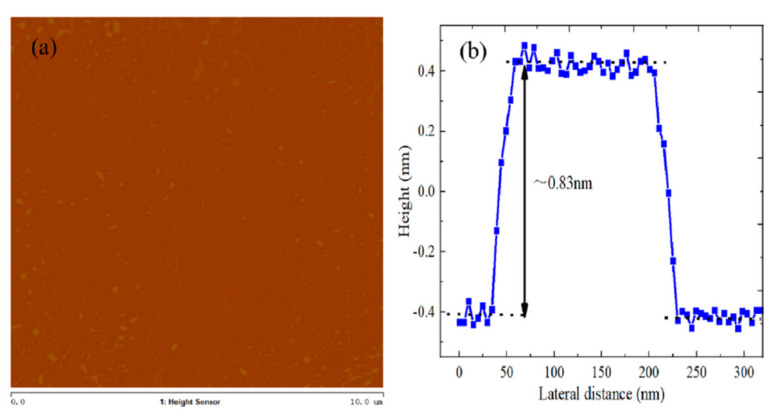
(**a**) AFM image and (**b**) height profile of WS_2_ material.

The principle behind AFM is that a micro cantilever can measure the induction of a four-quadrant detector, and the force between tiny probe tips and surface atoms can be amplified to the point of detection [27]. Therefore, the resolution of AFM can reach the atomic level. The layer number and thickness of WS_2_ material can be characterized and determined by AFM. In Figure 4a, WS_2_ material has regular morphology and a relatively flat surface, but there are also some fine particles on the surface of the film, which can be explained by the presence of a residual tungsten source. The height difference between the substrate surface and the WS_2_ crystal film surface is 0.83 nm, which can be considered as monolayer WS_2_ material, as shown in Figure 4b.

### 3.2. Test Characterization of WS_2_/h-BN Vertical Heterostructure Material

**Figure 5:**
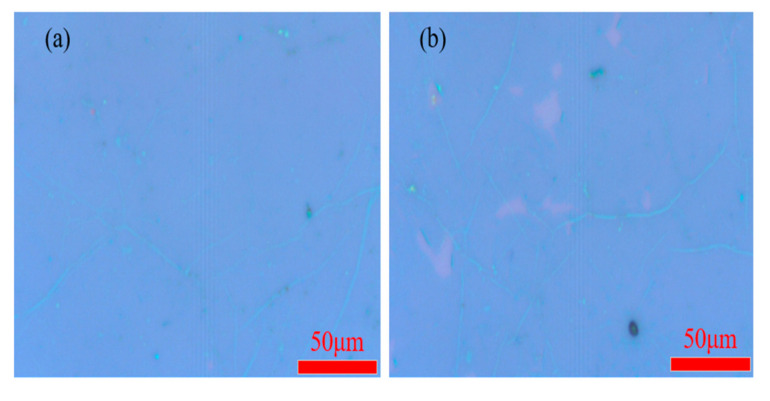
Optical microscope image of WS_2_/h-BN vertical heterostructure material. (**a**) position 1; (**b**) position 2.

The size and morphology of layered material can be observed directly by optical microscope. Meanwhile, a simple estimate of the layer number can be achieved by the different contrast under a microscope. The morphology of WS_2_/h-BN vertical heterostructure material can quickly be characterized by an optical microscope. In Figure 5, WS_2_/h-BN vertical heterostructure material on SiO_2_/Si substrate has better contrast under the position 1 and position 2, and the crystallinity and thickness can further be determined by optical contrast and Raman spectroscopy.

**Figure 6:**
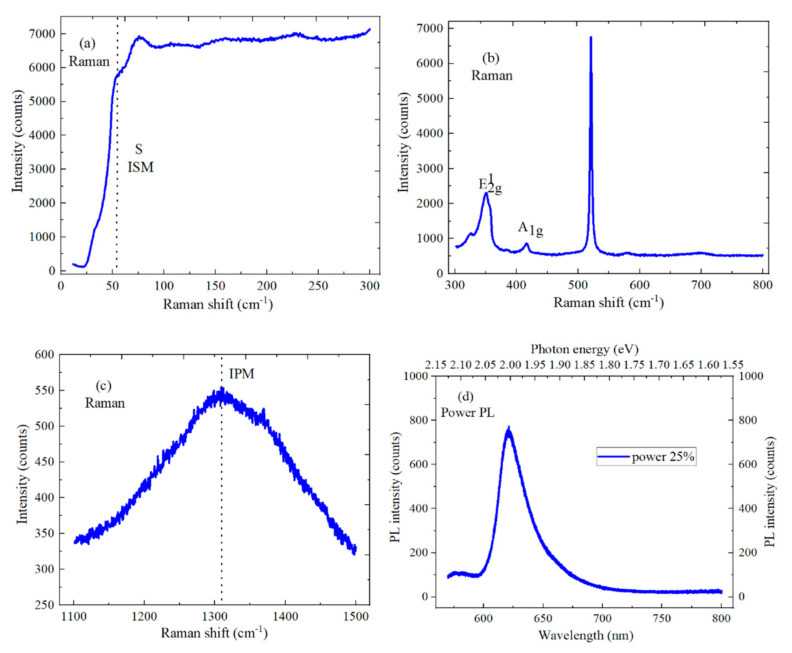
Spectral characteristics of WS_2_/h-BN heterostructure material. (**a**) ISM mode characteristic peak of h-BN material; (**b**) Raman spectrum of WS_2_ material; (**c**) IPM mode characteristic peak of h-BN material; (**d**) Photoluminescence (PL) spectrum of WS_2_/h-BN heterostructure material.

Photons excite inelastic collisions when a 532 nm laser is irradiated on the surface of a material, and this material’s information can be obtained by collecting and analyzing these photons. The 532 nm laser wavelength is used to explore the optical properties and energy band structure of WS_2_/h-BN vertical heterostructure material. A Raman process in h-BN material is non-resonant, which requires longer integration time and extra care. Figure 6a,c show that the ultra-low frequency and high-frequency lines are 52.5 cm^−1^ and 1366 cm^−1^ and correspond to interlayer shear mode (ISM) and in-plane mode (IPM), respectively. There is a massive difference in intensity between ISM and IPM modes, this being the sign of energy difference between the interlayer interaction and in-plane atomic interaction. The h-BN material can decrease the charge disorder and the scattering of charged impurities. In Figure 6b, the E^1^_2g_ in-plane vibration and A_1g_ out-of-plane vibration modes’ characteristic peaks of monolayer WS_2_ material are 351.2 cm^−1^ and 416.2 cm^−1^, respectively. The frequency difference between E^1^_2g_ and A_1g_ can be indicated as the layer number of 2D material, and the wavenumber difference is 65 cm^−1^, which is an indicator of good monolayer crystal. The intensity difference between the two vibration peaks of the monolayer WS_2_ material is relatively large, and the peak value of the E^1^_2g_ vibration mode is significantly larger than that of the A_1g_ vibration mode. Photoluminescence (PL) spectroscopy is a non-contact and non-destructive characterization method, which is used to detect and judge the band gap between materials. The band gap changes with the layer number of the 2D material, which can determine the layer number and band gap. In Figure 6d, a 532 nm laser is used to test and characterize the WS_2_/h-BN vertical heterostructure material, the peak position of the strongest excitation peak is 620.9 nm, and the corresponding energy band gap is 2 eV. The intensity of the PL spectrum is related to the quality and layer number of the material, and the high luminous intensity indicates the high quality of the WS_2_/h-BN heterostructure material. Meanwhile, the PL spectrum of WS_2_ material also shows sharp exciton transition and higher PL intensity, which can be explained by the dielectric shielding effect of h-BN material. There is strain and charge rearrangement at the interface of the WS_2_/h-BN heterostructure material.

### 3.3. Preparation and Test Characterization of FET Device with WS_2_/h-BN Vertical Heterostructure Material 

**Figure 7:**
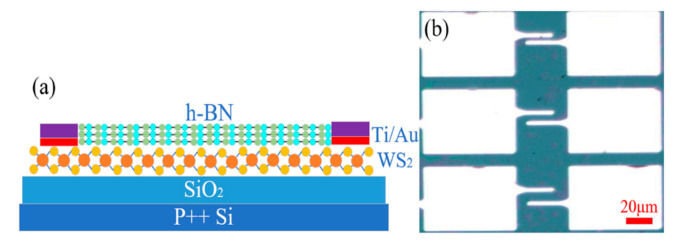
(**a**) Schematic diagram and (**b**) optical microscope image of FET array devices with WS_2_/h-BN vertical heterostructure material.

The adsorbed molecules on the surface of the semiconductor material would cause the hysteresis characteristics of 2D nano-electronic devices. Figure 7a shows the schematic diagram of the device, and h-BN material is used as an encapsulation layer to decrease the capture and release of interface charge, which can improve the electrical performance. In Figure 7b, Ti/Au (20 nm/70 nm) bimetals were deposited on WS_2_/h-BN vertical heterostructure material by standard electron beam lithography and electron beam evaporation processes, and the deposition rate and pressure are 1 Å/s and 10^-7^ Torr, respectively. Ti metal has strong adhesion to the SiO_2_/Si substrate, but it also causes poor semi-metal contact. The length (L) dimension of the device is 20 μm, and the width (W) dimensions are designed to be 5 μm, 10 μm and 15 μm, respectively. 

**Figure 8:**
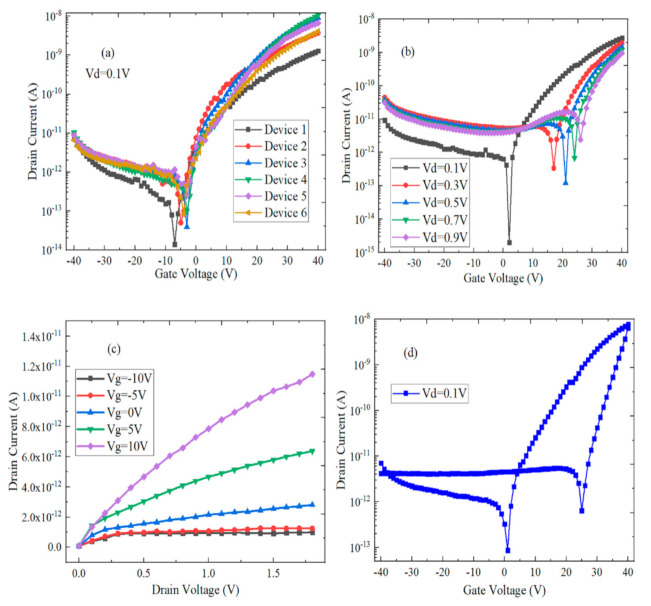
Electrical performance test of WS_2_/h-BN FET device. (**a**) Transmission characteristic curves (Id-Vg) of different devices; (**b**) Transmission characteristic curves (Id-Vg) under different drain voltages (Vd); (**c**) The output characteristic curve (Id-Vd) under different gate voltages (Vg); (**d**) Bidirectional transmission characteristic curve.

The intensity and magnitude of the current can be adjusted and controlled by the electric field effects in FET devices. Figure 8a shows the transfer characteristic curves of different FET devices with WS_2_/h-BN heterostructure material. As bipolar FETs, the branch current of n-type is much larger than that of p-type in the above devices. The gate voltage (Vgs) of the minimum source-drain current (Ids) value was around −5 V. Ids increases significantly by 5 orders while Vgs increases from −5 V to 40 V, which behaves like the n-type FET. When Vgs decreases from −5 V to −40 V, Ids increases monotonically, and the devices behave as p-type FET, which indicates that Fermi level can be adjusted to near valence band. On-off ratio and mobility are the main parameters by which to measure the capability of digital logic devices. The off-state leakage and on-state currents of device 3 can reach 3.9 × 10^−14^ A and 9.14 × 10^−9^ A when the gate voltage is selected as −3 V and 40 V, respectively. Additionally, the switching ratio is calculated to be 2.34 × 10^5^, which can implement the logic operations. The following is the calculation formula of device mobility:

(1) $$\mu =\frac{{\mathrm{dI}}_{\mathrm{ds}}}{{\mathrm{dV}}_{\mathrm{gs}}}\frac{L}{W\left(\varepsilon_{0}\varepsilon_{r}/d\right)V_{\mathrm{ds}}}$$

In Equation (1), μ, L, W, ε_0_, ε_r_ and d represent the mobility, length, width, vacuum dielectric constant, relative dielectric constant of SiO_2_ and thickness of SiO_2_, respectively. The mobility of device 3 is 3.341 cm^2^/Vs. The transfer characteristic curves of WS_2_/h-BN FET under different drain voltages (Vds) are shown in Figure 8b. The gate voltage position of minimum Ids value shifts to the right as Vds increases, off-state current increases, and the corresponding switching ratio decreases. Figure 8c shows the output characteristic curves of WS_2_/h-BN FET and shows that the relationship of Ids to Vds is nonlinear at different Vgs. The larger Schottky barrier and contact resistance exist between WS_2_/h-BN heterostructure material and the Ti/Au metal electrode interface. It can be found by comparing the transfer characteristic and the output characteristic curves of WS_2_/h-BN FET, where the Ids of the output characteristic curve is much smaller than that of the transfer characteristic curve, which is caused by the hysteresis characteristics of the FET device. The transfer characteristic curve of WS_2_/h-BN FET is shown in Figure 8d, where the obvious hysteresis phenomenon exists within the atmospheric environment. The specific measurement method is that Vds is set to 0.1 V, Vgs is first scanned forward from −40 V to 40 V and then Vgs is scanned backward from 40 V to −40 V. The device behaves as the bipolar FET when Vgs scanning is in the forward direction, the minimum Ids value is located at Vgs = 1 V and the P-type branch current is very small. When Vgs scanning is in the reverse direction, the minimum Ids value is at Vgs = 25 V and the voltage offset value is 24 V. Meanwhile, in the branch where Vgs is greater than 0 V, threshold voltage shifts to the right and Ids increases significantly during reverse scanning. This is because the charge capture and release process and the scattering of charged impurities can be significantly decreased by h-BN material [28].

## 4. Conclusions

In this paper, WS_2_/h-BN vdWs vertical heterostructure material was prepared using the CVD and wet transfer methods. Raman spectrometer, AFM, SEM, XPS and EDX were used to test and characterize WS_2_/h-BN vdWs vertical heterostructure material. Meanwhile, FET devices were fabricated on WS_2_/h-BN vdWs vertical heterostructure material by standard electron beam lithography and electron beam evaporation processes, and the electrical performance of FETs was also tested and analyzed. High-quality h-BN material can effectively decrease the charge capture and release process between WS_2_ material and gate oxide dielectric, and the scattering of charged impurities is also significantly reduced. The switching ratio of the WS_2_/h-BN FET device can reach 10^5^, and the mobility is 3.341 cm^2^/Vs, which meets the basic requirements of logic operations. Therefore, our research provides guidance and assistance in the application of vertical heterostructure material within the fields of electronics and optoelectronics.
